# Health- and Taste-Related Attitudes Associated with Dietary Patterns in a Representative Sample of Polish Girls and Young Women: A Cross-Sectional Study (GEBaHealth Project)

**DOI:** 10.3390/nu10020254

**Published:** 2018-02-23

**Authors:** Joanna Kowalkowska, Marta Lonnie, Lidia Wadolowska, Jolanta Czarnocinska, Marzena Jezewska-Zychowicz, Ewa Babicz-Zielinska

**Affiliations:** 1 Department of Human Nutrition, University of Warmia and Mazury in Olsztyn, Sloneczna 45F, 10-718 Olsztyn, Poland; joanna.kowalkowska@uwm.edu.pl (J.K.), lidia.wadolowska@uwm.edu.pl (L.W.); 2 Institute of Human Nutrition and Dietetics, Poznan University of Life Sciences, Wojska Polskiego 28, 60-637 Poznan, Poland; jolanta.czarnocinska@up.poznan.pl; 3 Department of Organization and Consumption Economics, Warsaw University of Life Sciences, Nowoursynowska 159 C, 02-776 Warsaw, Poland; marzena_jezewska_zychowicz@sggw.pl; 4 Faculty of Physiotherapy and Health Sciences, Gdansk Management College, Pelplinska 7, 80-335 Gdansk, Poland; zielarz.47@wp.pl

**Keywords:** attitudes, dietary patterns, adolescents, girls, health, taste, food, HTAS

## Abstract

Attitudes can be predictors of certain health-related behaviours. The attitudes of young females towards health and taste have not been yet fully examined and their associations with dietary behaviours remain unclear. The aim of the study was to investigate if attitudes are associated with dietary patterns in a representative sample of Polish girls. The study population consisted of 1107 girls, aged 13–21 and living in Poland. Attitudes were assessed using the Health and Taste Attitudes Scale (HTAS) and categorised as negative, neutral or positive. Dietary data was obtained using a Food Frequency Questionnaire. Dietary patterns (DPs), derived previously with a Principal Component Analysis (PCA), were ‘Traditional Polish’, ‘Fruit and vegetables’, ‘Fast food and sweets’ and ‘Dairy and fats’. The associations between attitudes and DPs were assessed using Spearman’s correlation coefficients and logistic regression. The reference group were girls with neutral attitudes. Odds ratios (ORs) were adjusted for age, socioeconomic status (SES), and body mass index (BMI). The correlations between attitudes and DPs ranged from −0.28 for attitudes towards health and ‘Fast food and sweets’ and ‘Traditional Polish’ DPs to 0.33 for attitudes towards health and the ‘Fruit and vegetables’ DP (*p* < 0.05). In the logistic regression analysis, the strongest associations within health-related HTAS subscales were observed between negative attitudes towards natural products and the ‘Fast food and sweets’ DP (OR: 10.93; 95% CI: 3.32–36.01) and between positive attitudes towards health and the ‘Fruit and vegetables’ DP (OR: 5.10; 3.11–8.37). The strongest associations within taste-related HTAS subscales were observed between positive attitudes towards craving for sweet foods and the ‘Traditional Polish’ DP (OR: 1.93; 1.43–2.61) and between positive attitudes towards using food as a reward and the ‘Dairy and fats’ DP (OR: 2.08; 1.22–3.55) as well as the ‘Fast food and sweets’ DP (OR: 2.07; 1.14–3.74). Positive attitudes towards health were associated with a pro-healthy dietary pattern characterised by the consumption of fruit and vegetables, while negative attitudes towards natural products as well as a strong craving for sweets and using food as a reward were associated with less healthy dietary patterns. To improve the dietary habits of girls and young women, positive attitudes towards health should be strengthened and supported by emphasizing the sensory values of pro-healthy foods.

## 1. Introduction

Numerous studies have reported the ‘westernisation’ of diets among young people in both developed and developing countries [1,2,3]. This is a matter of concern due to negative health outcomes associated with an excessive consumption of refined sugars, salt and saturated fats specific to this unhealthy trend [4,5]. Inadequate nutrition can have an impact not only on physical [6,7] but also mental health [8] and can subsequently affect young peoples’ growth and development. Furthermore, eating habits established during this crucial stage in life may track into adulthood [9], increasing the risk of developing non-communicable diseases in later life [10]. Finally, the impact of the mother’s diet on foetal and child health cannot be underestimated [11], making adolescent girls and young women a population of particular importance. Therefore, targeted and effective interventions are needed to correct and prevent the establishment of poor dietary habits. This can be achieved through building an understanding of what influences food choices and identifying potentially modifiable factors.

Since attitudes can influence and, to some extent, predict dietary behaviour [12], they are a variable worth more in-depth investigation. Attitudes are defined as an “evaluation (favourable or unfavourable) of performing a behaviour” [13] which may vary depending on age, gender, socioeconomic status (SES), body composition and lifestyle factors [12,14]. Pro-healthy attitudes tend to be more prevalent in older age groups, females, people with higher SES, those with a higher education level and also in individuals with a lower body mass index (BMI) [12,15,16]. These results indicate that attitudes differ in each group of people and therefore, each subpopulation requires separate and specific analysis. Although a range of data describing the attitudes towards food or healthy eating in adults is available [12,15,16,17], the population of adolescent girls and young women has not been examined in full.

Research methods used in investigating the association between attitudes and actual dietary behaviour found in previous studies have certain limitations. A number of studies have examined the associations between attitudes towards health and taste in relation to the consumption of single foods or nutrients (i.e., fruits, vegetables, high-fat foods or organic foods), indicating that people with positive attitudes are more likely to make healthier dietary choices than people with negative attitudes [18,19]. Although this is a valuable observation, it has to be noted that diet has multidimensional characteristics and, therefore, a more comprehensive approach in the analysis is needed to reflect the overall dietary behaviour, rather than providing information on single exposures [20]. Analysis of dietary patterns (DPs), as a more holistic and informative dietary approach, may help to formulate dietary advice which can then be used to plan appropriate nutrition education programmes.

To the authors’ knowledge, no study has yet investigated the links between attitudes and empirically derived DPs in young females. To fill this gap in the literature, the authors sought to investigate if attitudes are associated with DPs in a representative sample of Polish girls and young women.

## 2. Methods

### 2.1. Ethics Statement

The study was approved by the Bioethics Committee of the Faculty of Medical Sciences, University of Warmia and Mazury in Olsztyn, in Poland on the 17 June 2010 (Resolution No. 20/2010). Written consent to participate in the study was collected from women over 18-years-old and parents or legal guardians of girls under 18.

### 2.2. Study Design and Sample Collection

The data originated from the GEBaHealth Study—a cross-sectional study of dietary behaviours of Polish girls and young women, aged 13–21 years. The sampling protocol has been previously described [21,22]. In brief, 2104 participants were randomly selected from the general population of female residents born between 1991–1999, using the PESEL (Universal Electronic System of Population Register) database. Because access to a large, nation-wide sample was unfeasible for a small research team, data collection was outsourced to a third party specialising in large, national data collection (Centre for Public Opinion Research, CBOS). To ensure the representativeness of the national sample, the population was stratified proportionally to the general population, regarding the national region and location (city, town, rural). In total, 65 mutually exclusive strata were created, and participants were selected at random from each stratum.

The data regarding attitudes towards health and taste, dietary behaviour and socioeconomic status were collected using the closed-question questionnaire in October and November 2012. The study was conducted in the respondents’ homes by well-trained interviewers using the Computer-Assisted Personal Interviewing (CAPI) technique. Due to reasons such as respondent’s absence or respondent or parent/guardian refusal, 997 interviews were not possible to conduct. The response rate was 52.6%, and the study involved 1107 girls and young women. Due to missing data regarding height (*n* = 3) and body weight (*n* = 15), BMI was calculated for a smaller sample (*n* = 1092). To obtain a nationally representative sample, analyses were conducted with survey weights, correcting the non-response data and adjusting for unequal selection. Survey weights were calculated using mathematical modelling and included three weighting variables: age (three categories), place of residence (rural/urban) and national region. More details regarding of the study have been described in previous papers [21,22].

### 2.3. Attitudes towards Health and Taste

Attitudes towards health and taste were studied using Health and Taste Attitude Scales (HTAS) [23]. The HTAS included six subscales of which three subscales were health-related and three subscales referred to taste. The health scale consisted of three subscales: general health interest (eight statements), light product interest (six statements) and natural product interest (six statements). The taste scale consisted of three subscales: craving for sweet foods (six statements), using food as a reward (six statements) and pleasure (six statements). Respondents’ answers were recorded using a seven-point Likert scale, which is a common approach to measuring attitudes by placing the responses in rank order and is widely used in behavioural research. In our study, respondents were asked to indicate to what extent they agreed with each statement by choosing one of seven answers—from ‘strongly disagree’ (0 points) to ‘strongly agree’ (6 points). For example, one of the statements within the ‘light product interest’ subscale was as follows: ‘I believe that eating light products keeps one’s body in shape’. Assigning a high number of points to this and the remaining five statements within this subscale indicated that the respondent highly agreed with the statements and had a positive attitude towards light products. A complete list of statements within each HTAS subscale can be found in the Supplementary Material (Table S1). The subscales of HTAS had equal numbers of positively and negatively worded statements. The statements including negation were re-coded. The points obtained by every respondent in each subscale were then summed. The total scale range was 0–48 points for ‘General health interest’ and 0–36 for the remaining five subscales. Respondents were assigned into one of three categories—negative, neutral or positive attitude—towards health and taste within each of the six subscales of HTAS (Table 1). The cut-offs for category assignment (negative/neutral/positive) were 1/3 and 2/3 of the total scale range.

### 2.4. Dietary Data

The methods of collecting dietary data have been previously described in detail [21,22]. Briefly, respondents’ diets were studied using data from three short, validated food frequency questionnaires (FFQs): Block Screening Questionnaire for Fruit/Vegetable/Fibre Intake (BSQFVF) [24], Block Screening Questionnaire for Fat Intake (BSQF) [24] and the Food Intake Variety Questionnaire (FIVeQ) [25]. The Block questionnaires were used after modification and adjustment to the Polish diet and language [26]. The BSQFVF and BSQF provided data regarding the consumption frequency of 22 dietary items (each item with five response categories): fruit or vegetable juices; fruit (without juices); green salad; potatoes; beans; prepared vegetables (e.g., cooked, preserved or marinated, excluding beans); high-fibre or bran cereal; wholegrain bread; white bread (including biscuits, muffins); hamburgers or cheeseburgers; red meats (e.g., pork, beef); fried chicken; hot dogs or frankfurters; luncheon meats or bacon or fatty sausages; salad dressings or mayonnaise (not diet); margarine or butter; eggs; cheese or cheese spread; whole milk; French fries or potato chips or corn chips or popcorn; ice cream; doughnuts or pastries or cake or cookies. The FIVeQ provided data regarding the consumption frequency of 60 food items. Respondents expressed their consumption frequency in two categories (either ‘yes’ or ‘no’). The answer ‘yes’ meant that the food item had been consumed during the last week in an amount exceeding two tablespoons, seven bread slices or seven glasses, depending on the product type. Next, the 60 food items were subdivided into eight exclusive food groups, as follows: cereals and potatoes (six food items); dairy products (four food items); meats, fish and eggs (12 food items); vegetables (14 food items); fruit (eight food items); fats (six food items); sweets and snacks (four food items); beverages (without alcohol) (six food items.). Food intake variety within each of the eight food groups was expressed as the number of food items eaten per week. These eight food groups served as dietary items that were included in the principal component analysis (PCA). In total, the three questionnaires provided data regarding 30 dietary items, sufficient to capture individuals’ habitual diets through dietary pattern derivation (see Supplementary Material, Tables S2 and S3).

### 2.5. Confounding Factors

In assessing the association of attitudes towards health and taste on dietary patterns, the following confounders were taken into account: age (in years), socioeconomic status (SES), and weight status measured by BMI; the assessment methods were completed as previously reported [21,22]. Briefly, four variables were included in the SES index: mother’s education (three categories), father’s education (three categories), economic status (three categories) and description of household (five categories). For all categories within each of the SES variables, ranks were assigned—the higher the values, the more favourable the SES. The SES index was calculated as the sum of ranks of four variables. All variables which were a part of the SES assessment were standardised to have a mean of 0 and a standard deviation of 1. This process enabled the comparison of data received in different formats across SES categories. Self-reported height and weight were collected. To avoid bias, the obtained values were corrected using regression equations for adolescents, developed in a previous youth study [27]. Body mass index (BMI, kg/m^2^) was then calculated, and BMI categories were assigned in accordance with the International Obesity Task Force (IOTF) BMI cut-off points: age-sex specific cut-offs for girls 13–18 years old and cut-offs for girls at age 18 for those who were over 18 [28].

### 2.6. Statistical Analysis

The methods of deriving dietary patterns have been previously described in detail [21,22]. In brief, PCA has been applied to derivate DPs. Varimax normalised rotation and the eigenvalues of at least 1.00 were used. The total variance explained was 33.9%. Pattern labelling was based on a thorough analysis of the components included in each of the patterns (dietary items with factor loadings equal to 0.40 or higher). Concise names of the patterns represented the most prominent features and were labelled, as follows:

- ‘Traditional Polish’ (DP1) loading was based on white bread frequency consumption (factor-loading 0.65), meats/fish/eggs intake variety (0.60), potato frequency consumption (0.52), red meat frequency consumption (0.51), margarine or butter frequency consumption (0.45), fried chicken frequency consumption (0.42), fat intake variety (0.40), wholegrain bread frequency consumption (−0.48; the reverse relationship);
- ‘Fruit and vegetables’ (DP2) loading was based on vegetable intake variety (0.60), green salad frequency consumption (0.57), fruit frequency consumption (0.55), prepared vegetable frequency consumption (0.55), fruit intake variety (0.54) and bean frequency consumption (0.45);
- ‘Fast food and sweets’ (DP3) loading was based on French fries or potato chips or corn chips or popcorn frequency consumption (0.71), hamburger or cheeseburger frequency consumption (0.60), ice cream frequency consumption (0.52), doughnut, pastry, cake or cookie frequency consumption (0.50), sweets and snacks intake variety (0.47) and salad dressing or mayonnaise (not diet) frequency consumption (0.42);
- ‘Dairy and fats’ (DP4) loading was based on: cereal and potato intake variety (0.56), dairy product intake variety (0.54), cheese or cheese spread frequency consumption (0.54), whole milk frequency consumption (0.49), margarine or butter frequency consumption (0.45), and fat intake variety (0.43) (see: Supplementary Material, Table S2) [21,22].

The variance explained by each pattern was 14.5% (DP1), 9.0% (DP2), 5.6% (DP3) and 4.8% (DP4).

Respondents were divided into three categories within each DP based on score tertile distribution. The association between attitudes towards health and taste and DPs was assessed using (i) Spearman’s correlation coefficients; (ii) percentage distribution and (iii) logistic regression analysis.

The three methods of statistical analysis used in the current study served to strengthen the statistical conclusion validity. The normality of variables was checked by a Kolmogorov–Smirnov test [29]. To calculate correlation coefficients, the sum of points for each of the HTAS subscales (after recoding) and factor loadings for DP as a continuous variable were used. The odds ratios (ORs) were adjusted for confounding factors: age (in years), SES index (as a continuous variable) and BMI (as a categorical variable, due to the age-related heterogeneity of the sample: under and over 18-years-old). The reference groups were respondents in the lower tertile of DPs and respondents with neutral attitudes towards health and taste (OR = 1.00). Ninety-five percent confidence intervals (95% CI) for ORs were calculated.

All analyses were conducted with survey weights to correct non-response data during the sample collection. The chi-square test and Wald’s statistics were used to compare all characteristics, and *p* < 0.05 was considered significant. The statistical analysis was carried out using STATISTICA software (version 12.0 PL; StatSoft Inc., Tulsa, OK, USA; StatSoft, Kraków, Polska).

## 3. Results

### 3.1. Sample Characteristics

Characteristics of the study participants are presented in Table 2. Nearly three-quarters of females had neutral attitudes in each of the three health-related subscales (range: 73.2–74.8%). About one-fifth of girls had a positive attitude towards natural products (20.9%) and a similar percentage of girls had a negative attitude towards light products (20.1%). Considering taste-related attitudes, nearly half of the girls had positive attitude towards craving for sweet foods (48.9%) and almost one-third had a positive attitude towards food as a source of pleasure (29.1%). Additional data can be found in the Supplementary Materials: (1) distribution (%) of dietary patterns depending on attitudes towards health and taste in girls and young women (Table S4) and (2) the sample distribution (%) of dietary patterns depending on attitudes towards health and taste in girls and young women across BMI categories (Table S5).

### 3.2. Correlations between Attitudes and Dietary Patterns

**Figure 1:**
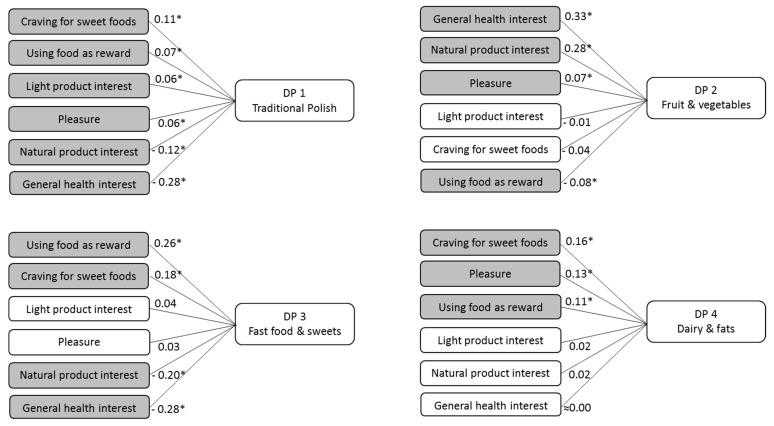
Spearman’s correlation coefficients for attitudes towards health and taste and dietary patterns (DPs) of girls and young women, the GEBaHealth Study (*n* = 1107); All data adjusted for survey weights. Statistically significant: * *p* < 0.05.

Relationships between attitudes and DPs, obtained using Spearman’s correlation coefficient, ranged from 0.06 to 0.33 (*p* < 0.05) for significant positive correlations and −0.08 to −0.28 (*p* < 0.05) for significant negative correlations (Figure 1).

‘General health interest’ was positively correlated with the ‘Fruit and vegetables’ DP (*r* = 0.33), and negatively correlated with the ‘Traditional Polish’ (*r* = −0.28) and ‘Fast food and sweets’ (*r* = −0.28) DPs. ‘Light product interest’ was positively correlated with ‘Traditional Polish’ (*r* = 0.06). ‘Natural product interest’ was positively correlated with the ‘Fruit and vegetables’ (*r* = 0.28), and negatively correlated with the ‘Fast food and sweets’ (*r* = −0.20) and ‘Traditional Polish’ (*r* = −0.12) DPs. ‘Craving for sweet foods’ was positively correlated with three dietary patterns: ‘Fast food and sweets’ (*r* = 0.18), ‘Dairy and fats’ (*r* = 0.16) and ‘Traditional Polish’ (*r* = 0.11). ‘Using food as reward’ was positively correlated with the ‘Fast food and sweets’ (*r* = 0.26), ‘Dairy and fats’ (*r* = 0.11) and ‘Traditional Polish’ (*r* = 0.07) DPs, and negatively correlated with the ‘Fruit and vegetables’ (*r* = −0.08) DP. ‘Pleasure’ was positively correlated with the ‘Dairy and fats’ (*r* = 0.13), ‘Fruit and vegetables’(*r* = 0.07) and ‘Traditional ‘Polish’ (*r* = 0.06) DPs.

### 3.3. Associations Attitudes and Dietary Patterns

The odds ratios for positive and negative attitudes across tertile categories of DPs are presented in Table 3.

#### 3.3.1. Health-Related Attitudes

Girls with a positive attitude towards health were more likely to adhere to middle and upper tertiles of the ‘Fruit and vegetables’ DP (OR: 2.98; 95% CI: 1.77–4.99 and 5.10; 3.11–8.37), and less likely to adhere to the middle and upper tertiles of the ‘Fast food and sweets’ DP (OR: 0.54; 0.37–0.77 and 0.32; 0.21–0.49), the middle and upper tertiles of the ‘Traditional Polish’ DP (OR: 0.35; 0.23–0.52 and 0.40; 0.27–0.59) and the middle tertile of the ‘Dairy and fats’ DP (OR: 0.48; 0.32–0.72). Girls with a negative attitude towards health were more likely to adhere to the middle and upper tertiles of the ‘Fast food and sweets’ DP (OR: 2.04; 1.18–3.55 and 3.39; 2.00–5.75) and the middle and upper tertiles of the ‘Traditional Polish’ DP (OR: 1.93; 1.14–3.27 and 2.63; 1.55–4.45), and less likely to adhere to the upper tertile of the ‘Fruit and vegetables’ DP (OR 0.33; 0.20–0.56). Girls with a positive attitude towards natural products were more likely to adhere to the middle and upper tertiles of the ‘Fruit and vegetables’ DP (OR: 1.59; 1.10–2.29 and 2.23; 1.57–3.18), and less likely to adhere to the upper tertile of the ‘Traditional Polish’ DP (OR: 0.62; 0.44–0.87) and the middle and upper tertiles of the ‘Fast food and sweets’ DP (OR: 0.63; 0.45–0.87 and 0.49; 0.35–0.70). Girls with a negative attitude towards natural products were more likely to adhere to the upper tertile of the ‘Fast food and sweets’ DP (OR: 10.93; 3.32–36.01) and the middle and upper tertiles of the ‘Traditional Polish’ DP (OR: 2.86; 1.24–6.59 and 2.51; 1.03–6.14). No significant associations were found between a positive attitude towards light products and any of the DPs. Girls with a negative attitude towards light products were less likely to adhere to the middle tertiles of the ‘Traditional Polish’ DP (OR: 0.68; 0.48–0.97) and the ‘Fruit and vegetables’ DP (OR: 0.65; 0.45–0.92).

#### 3.3.2. Taste-Related Attitudes

Girls with a positive attitude towards craving for sweet foods were more likely to adhere to the upper tertile of the ‘Traditional Polish’ DP (OR 1.93; 1.43–2.61), the middle and upper tertiles of the ‘Fast food and sweets’ DP (OR: 1.50; 1.12–2.00 and 1.84; 1.38–2.45) and the upper tertile of the ‘Dairy and fats’ DP (OR 1.57; 1.18–2.09). Girls with a negative attitude towards craving for sweet foods were less likely to adhere to the upper tertile of the ‘Dairy and fats’ DP (OR 0.48; 0.26–0.91). Girls with a positive attitude towards using food as a reward were more likely to adhere to the upper tertiles of the ‘Dairy and fats’ DP (OR: 2.08; 1.22–3.55) and the ‘Fast food and sweets’ DP (OR: 2.07; 1.14–3.74). Girls with a negative attitude towards using food as reward were less likely to adhere to the upper tertile of the ‘Dairy and fats’ DP (OR: 0.72; 0.53–0.96) and the middle and upper tertiles of the ‘Fast food and sweets’ DP (OR: 0.69; 0.52–0.92 and 0.35; 0.26–0.47). Girls with a positive attitude towards food as a source of pleasure were more likely to adhere to the middle tertile of the ‘Fruits and vegetables’ DP (OR: 1.59; 1.17–2.18). Girls with a negative attitude towards food as a source of pleasure were less likely to adhere to the middle tertile of the ‘Dairy and fats’ DP (OR: 0.27; 0.07–1.00).

## 4. Discussion

It was found that health- and taste-related attitudes are associated with dietary patterns in Polish girls and young women. In general, positive health-related attitudes were associated with a pro-healthy dietary pattern, while negative health-related attitudes and positive taste-related attitudes were associated with less healthy dietary patterns.

The associations between health- and taste-related attitudes and dietary patterns in Polish girls and young women were confirmed in all three analyses performed. The correlations between attitudes and dietary patterns were weak, but significant (max. *r* = 0.33), which is consistent with previous studies on attitudes and health-related behaviours [30,31]. Although all analyses confirmed the associations, slight differences in results were observed. These discrepancies might have been caused by the adjustment for potential confounders—possible to perform only in the logistic regression analysis. Because the latter analysis reflects the actual relationship between the variables more precisely by eliminating the effect of confounding factors, the discussion was focused on these results.

### 4.1. Attitudes as Predictors of a Pro-Healthy Dietary Pattern

Girls with a positive attitude towards health were over five times more likely to adhere to the ‘Fruit and vegetables’ DP and less likely to adhere to rather the unhealthy ‘Traditional Polish’ and unhealthy ‘Fast food and sweets’ DPs. Similarly, girls with a positive attitude towards natural products were over two times more likely to have a diet characterised by the consumption of fruit, vegetables and legumes.

These findings are consistent with previous studies in adult populations in which health interest was associated with healthier food choices, including a higher consumption of fruit and vegetables [17,18,32]. Health interest and natural product interest have been previously found to be higher in females than in males [23]. Fruit and vegetable consumption and naturalness of food are consistently reported as essential characteristics of healthy eating [33]. Across many countries, natural food products are perceived as those that are unprocessed, and without additives or pollution [34]. Furthermore, plant foods are more frequently reported as natural than animal products. An attitude is a complex concept, defined as an overall evaluation of an object based on a variety of information and including three components: cognitive, affective and behavioural [35,36]. Thus, positive attitudes towards both health and natural products, as reported by girls and young women, may result from their greater nutritional knowledge and awareness of diet–disease relationships. It has been shown that greater nutritional knowledge is associated with healthier dietary choices, although the strength of this association was rather weak [37,38,39]. A systematic review of the literature showed that nutritional knowledge most often was positively associated with a higher consumption of fruit and vegetables [39]. Similar associations were found for adults with a higher health interest [32]. Another study showed that nutrition knowledge alone was often not sufficient to improve the eating habits of young people [37]. The review by Taylor et al. [40] showed that food preferences appear to have a stronger impact on dietary behaviours than nutritional knowledge among young people. This, combined with perhaps a low motivation of some females towards healthy eating [16], could explain why not all, but less than half, of girls with a positive attitude towards natural products were in the upper tertile of the ‘Fruit and vegetables’ DP.

The association between perceiving food as a source of pleasure and the ‘Fruit and vegetable’ DP was relatively week and ambiguous—the result was significant for the middle, but not for the upper tertile of this DP characterised by consumption of fruit, vegetables and legumes. Sensory properties of food are one of the most important factors in food choices. For instance, Kumar et al. [41] observed that for high school students the main motivators of eating healthy were both the desire to be healthy and enjoying the taste of healthy foods. Nevertheless, in contrast to fruit, vegetables are rarely perceived as enjoyable foods by children and young adults [42,43]. Recent trends that are popular in Poland for vegetable preparation—serving vegetables with various dips, dressings and in the form of stir-fries—may have contributed to improving the palatability of vegetables among girls. This provides an optimistic outlook for young females’ perceptions of vegetables, suggesting that given the right attitude and perhaps an attractive preparation form, these foods can be consumed as an enjoyable part of a diet.

No clear associations were found in terms of expressing interest in light products, which was a surprising result considering the commonly observed weight control behaviours among girls [44,45]. According to the HBSC study (2013–2014), among Polish girls, about one-quarter of 11-year-olds and more than one-third of 15-year-olds reported using a slimming diet [44]. It has been shown that the main factors for choosing healthy food were maintaining good health, taste and weight reduction [46]. Low calorie foods were perceived by young women as healthier and trendier, but also less tasty than high-energy foods [47]. This suggests the possibility of ambivalent feelings towards these products among females or indecisiveness in answering, which can be interpreted as a neutral attitude [48,49]. The relationship between attitude and behaviour has been previously shown to be stronger in people with lower ambivalence and more confidence in determining their attitude towards food products [48]. Perhaps the knowledge of girls about light products and their composition and impact on health was insufficient, and therefore, the associations between attitudes towards light products and dietary patterns remained statistically insignificant.

### 4.2. Attitudes as Predictors of Unhealthy Dietary Patterns

Girls with negative attitudes towards both health and natural products were more likely to adhere to unhealthy (‘Fast food and sweets’) and rather unhealthy (‘Traditional Polish’) DPs. For example, the chance of adherence to the upper tertile of the ‘Fast food and sweets’ DP among girls with a negative attitude towards natural products was nearly 11-fold higher than in girls with a neutral attitude towards such foods. However, it needs to be acknowledged that girls with a negative attitude towards natural products represented only a small percentage of our study sample, and nearly three-quarters of girls had a neutral attitude towards natural products, which may suggest insufficient knowledge about these products.

Negative or neutral attitudes towards natural products could be attributed to a busy lifestyle and the ‘westernisation’ of the diet observed in Poland [1,3]. The availability of healthy snacks (e.g., pre-packed ‘ready to eat’ fruit) and restaurants serving natural, healthy foods is still limited in Poland, particularly outside big cities. A previous study showed that higher consumption of high energy-dense foods was related to weak motivation towards healthy eating, noted especially in men and young adults [16]. Adolescents tend to highlight taste preferences, price, convenience, social aspects and advertisement as important factors in dietary choices [50]. For young people, these factors can be more relevant than health. Karimi-Shahanjarini and colleagues [51] found that in girls aged 12–15 years, the main motivation for unhealthy snack consumption was taste, followed by other important factors such as peer pressure, easy access to unhealthy snacks as well as limited availability and high price of healthy snacks. Teenage females have also highlighted that healthiness of food and the possible risk of disease in the future were not their first priorities in making food choices, but caring for a slim silhouette could alter their behaviours [51].

Preferences for fast foods, mainly based on taste and easy access to these products, influenced young people’s food choices and were indicated as one of the main barriers to healthy eating [52]. In adults, four factors in attitudes towards fast food were identified concerning the following issues: perceived convenience, fun and social aspects, perceived unhealthiness and dislike towards cooking [53]. However, only two of them (i.e., perceived convenience of fast food and dislike of cooking) were associated with frequency of fast food consumption. It was suggested that family support as well as greater availability of healthy, inexpensive and tasty snacks and meals at school and other social spaces may facilitate healthier food choices among young people [52].

Regarding taste-related attitudes, a significant association was found for ‘craving for sweet foods’ and less healthy dietary patterns. Girls with a positive attitude towards craving for sweets were around twice more likely to adhere to the ‘Traditional Polish’ and ‘Fast food and sweets’ DPs and over one and a half times more likely to adhere to the ‘Dairy and fats’ DP. Many studies have shown that taste is a more important factor in food choices than healthiness, especially for young people [17,54,55]. Craving for sweets was previously reported to be more common among females than males [56], with females perceiving sweet snacks (e.g., chocolate) as comfort foods [57]. Considering sweets as mood boosters was reported to be the main reason for continuing this dietary habit in adults, despite an awareness of the negative health consequences of excess sweets consumption [58].

The study also found that a positive attitude towards perceiving food as a reward was associated with the ‘Fast food and sweets’ and ‘Dairy and fats’ DPs. Females having such an attitude were over two-fold more likely to adhere to the upper tertiles of both DPs. These findings are in line with previous studies showing that attitudes related to the taste of food (i.e., craving for sweet foods, using food as a reward) were good predictors of unhealthy dietary choices [17]. As the main factors hindering healthy eating, young adults reported taste preferences, the convenience and time spent cooking meals, the price and availability of food, self-discipline as well as the influence of peers, media and advertising [54]. Similarly, other studies have shown that the main motives of food choices among university students are price, sensory attractiveness of food and mood [59]. It was shown that sensitivity to reward was positively associated with consumption of unhealthy snacks and sugar-sweetened beverages in teenagers [60]. Craving for high-calorie foods and using food as a reward or a means to regulate emotions can result in over-consumption of unhealthy foods or consuming excessive portions of food, which may lead to eating disorders and weight gain [60,61,62,63]. Negative emotions can lead to higher consumption of products perceived as comfort food, such as ice cream, chocolate and crisps [63].

Positive or negative emotions linked to past and current experiences of consuming food play an important role in shaping the affective component of attitude, which, to some extent, influences later behaviour [64]. It has been shown that repeated exposure and rewarding can effectively modify children's food preferences and improve the liking and consumption of unfamiliar and/or healthy foods, such as various types of vegetables [65,66,67]. Children like and prefer to consume more familiar products [63]. While for women, the main barriers towards healthy eating and meeting the fruit and vegetable recommendations were a lack of cooking skills, the taste of healthy foods, preparation time and a lack of willpower [68]. Perhaps teaching cooking focused on quick and easy-to-make meals based on vegetables, legumes and whole grains as well as providing a wider offering of healthier, but tasty, snacks at both school and home may be helpful in improving young females’ eating behaviours [42,52].

### 4.3. Strengths and Limitations

The strength of the current study is its large, nationally representative sample and, therefore, the results can be generalised to the population of girls and young women in Poland. Furthermore, in the assessment of the relationship between attitudes and dietary behaviours, the study used a logistic regression analysis and minimised the influence of other factors by controlling the odds ratios for three important confounders (i.e., age, socioeconomic status and BMI). Although this study did not provide data regarding the energy value of girls’ diets, the BMI was included as a measure of the energy balance of the human body [69,70].

The study is not without limitations. The cross-sectional design of the present study and data collection at a single point in time did not allow conclusions to be drawn about causality, but only on the associations between attitudes and behaviours. Secondly, the study relied on self-reported data, and the possibility of social desirability bias should be taken into account [71,72]. In the assessment of attitudes, a Likert scale was used, which, although commonly involved in research, may entail some interpretative difficulties. The results for the middle of the range of points should indicate a neutral attitude, but such results may also indicate lack of knowledge, indecisiveness of the respondent or the balancing of extreme responses to individual statements of the scale [49]. However, to minimise the potential bias, we have used validated questionnaires in studying both attitudes and dietary behaviours.

### 4.4. Implications for Research and Practice

Negative attitudes towards health and natural products and positive attitudes towards hedonistic aspects of food were associated with dietary patterns characterised by a high consumption of fast foods, sweets, snacks and white bread, potatoes, meat and fat. Positive attitudes towards both health and natural products were associated with a pro-healthy DP, characterised by the consumption of wide variety of raw and processed vegetables, fruit, and legumes, but the association between perceiving food as a source of pleasure and the ‘Fruit and vegetables’ DP was not clear. Regardless of the important factors that have been taken into account in the assessment of the relationship between attitude and dietary patterns, other factors may also have affected food choices, such as peer pressure, lack of time, mood or cigarette smoking [54,64,73,74], and their inclusion should be considered in future research. Further research could also consider the inclusion of ethnicity and racial background as potentially confounding factors. At the time of data collection, Poland was not a very ethnically and racially diverse country (less than 2% of the general population); thus, this potential confounder was considered to have a marginal effect on the observations.

The current results could introduce important information to consider, especially in developing countries and countries in transition affected by rapid diet westernisation and increasing prevalence of overweight and obesity, where effective health promotion policies are urgently needed to prevent the potential onset of diet-related diseases. Attitude modification should be recognised as a potentially important component of strategies improving dietary habits targeting girls and young women. Although the promotion of positive attitudes towards health in young females should remain a relevant element in public health interventions, the current results suggest that attitudes conducive to healthier food choices should be developed, not only based on informing about food healthiness, but, from the earliest years, also on emphasizing the sensory value of healthy food, and incorporating cooking classes focused on preparing tasty, quick and easy-to-prepare meals from vegetables or legumes may be crucial. In the authors' opinions, to increase the effectiveness of health education programmes targeted at young females, the focus should be placed not only on health-related consequences of an unhealthy diet, but also on its effect on appearance, e.g., skin, hair or body weight, known as an important motivation in changing the lifestyles of teenagers and young women.

## 5. Conclusions

Health- and taste-related attitudes are associated with dietary patterns in Polish girls and young women. A positive attitude towards health is associated with a pro-healthy dietary pattern, characterised by the consumption of fruit, vegetables and legumes, while a negative attitude towards natural products as well as strong craving for sweets and using food as a reward is associated with less healthy dietary patterns, characterised by the consumption of food rich in refined carbohydrates and saturated fats. Therefore, positive attitudes towards health may be used as a predictor of pro-healthy dietary patterns, and positive attitudes towards taste as a predictor of non-healthy dietary patterns of girls and young women.

Attitude modification should be considered to be an important component of nutrition interventions targeting girls and young women. The adoption of a setting-based approach should be considered. Previous studies have shown that long-term interventions in schools and workplaces have been shown to be feasible and effective tools in modifying dietary choices [75,76]. In this context, we can direct the recommendations from our research. To improve the dietary behaviours of girls and young women, positive attitudes towards health should be strengthened and supported by emphasizing the sensory values of pro-healthy foods.

## Figures and Tables

**Table 1:** Categories and scoring of attitudes within Health and Taste Attitudes Scale (HTAS) subscales.

Scale	Range (Points)	Attitudes (Points)		
		Negative	Neutral	Positive
Health-related subscales				
General health interest	0–48	0–15	16–32	33–48
Light product interest	0–36	0–11	12–24	25–36
Natural product interest	0–36	0–11	12–24	25–36
Taste-related subscales				
Craving for sweet foods	0–36	0–11	12–24	25–36
Using food as a reward	0–36	0–11	12–24	25–36
Pleasure	0–36	0–11	12–24	25–36

**Table 2:** Sample characteristics, the GEBaHealth Study (*n =* 1107) ^†^.

Sample Characteristics	*N*	%
**Age categories (years)**		
13–15	326	29.5
16–18	367	33.1
19–21	414	37.4
**Age** (years; mean (SD))	17.3 (2.6)	
**Body weight status ^‡^**		
Thinnest grade 3	0	0.0
Thinnest grade 2	5	0.5
Thinnest grade 1	105	9.7
Normal weight	849	77.7
Overweight	115	10.5
Obesity	18	1.6
**Attitudes towards health**		
General health interest		
Negative	123	11.1
Neutral	810	73.2
Positive	174	15.7
Light product interest		
Negative	222	20.1
Neutral	829	74.8
Positive	56	5.1
Natural product interest		
Negative	48	4.3
Neutral	828	74.8
Positive	231	20.9
**Attitudes towards taste**		
Craving for sweet foods		
Negative	69	6.2
Neutral	497	44.9
Positive	541	48.9
Using food as reward		
Negative	409	36.9
Neutral	606	54.8
Positive	92	8.3
Pleasure		
Negative	14	1.2
Neutral	771	69.7
Positive	322	29.1

^†^ All data adjusted for survey weights. SD—standard deviation. ^‡^ Body weight status—body mass index (BMI) categories determined according to International Obesity Task Force (IOTF) standards [28], i.e., for girls 13–18 years-old according to age-sex-specific BMI cut-offs and for girls >18-years-old according to cut-offs for girls at age 18; BMI sample size is smaller due to missing data (*n* = 1092).

**Table 3:** Adjusted odds ratios (OR; 95% CI) for dietary patterns depending on attitudes towards health and taste in girls and young women in the GEBaHealth Study (*n* = 1107) ^†^.

Attitudes towards Health and Taste	Dietary Patterns											
	‘Traditional Polish’			‘Fruit and Vegetables’			‘Fast Food and Sweets’			‘Dairy and Fats’		
	Bottom Tertile (*n* = 367)	Middle Tertile (*n* = 364)	Upper Tertile (*n* = 376)	Bottom Tertile (*n* = 364)	Middle Tertile (*n* = 367)	Upper Tertile (*n* = 376)	Bottom Tertile (*n* = 365)	Middle Tertile (*n* = 365)	Upper Tertile (*n* = 377)	Bottom Tertile (*n* = 366)	Middle Tertile (*n* = 365)	Upper Tertile (*n* = 376)
**General health interest**												
negative	Reference	1.93 * (1.14; 3.27)	2.63 *** (1.55; 4.45)	1.00	0.70 (0.47; 1.06)	0.33 **** (0.20; 0.56)	1.00	2.04 * (1.18; 3.55)	3.39 **** (2.00; 5.75)	1.00	0.73 (0.47; 1.13)	0.72 (0.46; 1.12)
neutral	1.00	1.00	1.00	1.00	1.00	1.00	1.00	1.00	1.00	1.00	1.00	1.00
positive	Reference	0.35 **** (0.23; 0.52)	0.40 **** (0.27; 0.59)	1.00	2.98 **** (1.77; 4.99)	5.10 **** (3.11; 8.37)	1.00	0.54 *** (0.37; 0.77)	0.32 **** (0.21; 0.49)	1.00	0.48 *** (0.32; 0.72)	0.76 (0.52; 1.11)
**Light product interest**												
negative	Reference	0.68 * (0.48; 0.97)	0.88 (0.62; 1.24)	1.00	0.65 * (0.45; 0.92)	0.94 (0.67; 1.32)	1.00	0.86 (0.61; 1.22)	0.82 (0.58; 1.16)	1.00	1.04 (0.73; 1.47)	1.04 (0.74; 1.48)
neutral	1.00	1.00	1.00	1.00	1.00	1.00	1.00	1.00	1.00	1.00	1.00	1.00
positive	Reference	0.86 (0.48; 1.54)	0.55 (0.29; 1.07)	1.00	0.86 (0.45; 1.65)	1.31 (0.71; 2.41)	1.00	0.70 (0.37; 1.32)	0.99 (0.55; 1.79)	1.00	1.04 (0.56; 1.94)	0.98 (0.49; 1.94)
**Natural product interest**												
negative	Reference	2.86 * (1.24; 6.59)	2.51 * (1.03; 6.14)	1.00	0.70 (0.36; 1.36)	0.49 (0.23; 1.03)	1.00	3.18 (0.87; 11.59)	10.93 **** (3.32; 36.01)	1.00	0.91 (0.44; 1.88)	1.17 (0.58; 2.36)
neutral	1.00	1.00	1.00	1.00	1.00	1.00	1.00	1.00	1.00	1.00	1.00	1.00
positive	Reference	0.74 (0.53; 1.02)	0.62 ** (0.44; 0.87)	1.00	1.59 * (1.10; 2.29)	2.23 **** (1.57; 3.18)	1.00	0.63 ** (0.45; 0.87)	0.49 **** (0.35; 0.70)	1.00	1.00 (0.64; 1.57)	1.19 (0.85; 1.67)
**Craving for sweet foods**												
negative	Reference	0.80 (0.46; 1.39)	0.70 (0.38; 1.29)	1.00	1.39 (0.78; 2.49)	0.93 (0.50; 1.70)	1.00	0.88 (0.52; 1.51)	0.55 (0.29; 1.03)	1.00	0.79 (0.46; 1.37)	0.48 * (0.26; 0.91)
neutral	1.00	1.00	1.00	1.00	1.00	1.00	1.00	1.00	1.00	1.00	1.00	1.00
positive	Reference	1.30 (0.97; 1.73)	1.93 **** (1.43; 2.61)	1.00	1.08 (0.81; 1.44)	0.78 (0.59; 1.04)	1.00	1.50 ** (1.12; 2.00)	1.84 **** (1.38; 2.45)	1.00	1.14 (0.85; 1.52)	1.57 ** (1.18; 2.09)
**Using food as reward**												
negative	Reference	0.78 (0.58; 1.04)	0.76 (0.56; 1.02)	1.00	1.09 (0.81; 1.46)	1.18 (0.88; 1.59)	1.00	0.69 * (0.52; 0.92)	0.35 **** (0.26; 0.47)	1.00	0.75 (0.56; 1.01)	0.72 * (0.53; 0.96)
neutral	1.00	1.00	1.00	1.00	1.00	1.00	1.00	1.00	1.00	1.00	1.00	1.00
positive	Reference	1.07 (0.63; 1.83)	1.07 (0.62; 1.85)	1.00	1.04 (0.64; 1.72)	0.67 (0.39; 1.17)	1.00	1.65 (0.91; 3.01)	2.07 * (1.14; 3.74)	1.00	0.95 (0.52; 1.76)	2.08 ** (1.22; 3.55)
**Pleasure**												
negative	Reference	0.41 (0.12; 1.33)	0.31 (0.08; 1.19)	1.00	0.91 (0.32; 2.60)	0.25 (0.05; 1.21)	1.00	1.61 (0.50; 5.13)	0.92 (0.24; 3.51)	1.00	0.27 * (0.07; 1.00)	0.35 (0.10; 1.30)
neutral	1.00	1.00	1.00	1.00	1.00	1.00	1.00	1.00	1.00	1.00	1.00	1.00
positive	Reference	1.16 (0.85; 1.59)	1.20 (0.87; 1.64)	1.00	1.59 ** (1.17; 2.18)	1.36 (0.99; 1.87)	1.00	1.10 (0.81; 1.50)	1.16 (0.86; 1.57)	1.00	0.86 (0.63; 1.18)	1.35 (1.00; 1.83)

^†^ All data adjusted for survey weights. OR—odds ratio adjusted for three variables: age (years), SES (continuous variable measured as socioeconomic status (SES) index which was calculated from four single components: mother’s education, father’s education, economic status, description of household), BMI (as categorical variable according to IOTF standards [28], i.e., for girls 13–18 years old according to age/sex specific BMI cut-offs and for girls >18-years-old according to cut-offs for girls at age 18). * *p* < 0.05; ** *p* < 0.01; *** *p* < 0.001; **** *p* < 0.0001 (Wald’s test).

## Supplementary Materials

The following are available online at http://www.mdpi.com/2072-6643/10/2/254/s1, Table S1: Statements within Health and Taste Attitudes Scales (HTAS) (Roininen et al., 1999). Table S2: Factor-loading matrix for the 4 major dietary patterns identified by principal component analysis. Table S3: Means with standard deviation (SD) for dietary characteristics across tertiles of dietary patterns. Table S4: Sample distribution (%) of dietary patterns depending on attitudes towards health and taste in girls and young women, the GEBaHealth Study (*n* = 1107). Table S5: Sample distribution (%) of dietary patterns depending on attitudes towards health and taste in girls and young women across BMI categories, the GEBaHealth Study (*n* = 1092).
