# Supplementary material for: Health- and Taste-Related Attitudes Associated with Dietary Patterns in a Representative Sample of Polish Girls and Young Women: A Cross-Sectional Study (GEBaHealth Project)
Source: Nutrients. 2018 Feb 23;10(2):254. doi: 10.3390/nu10020254 (PMC5852830; doi:10.3390/nu10020254)
Supplement: Supplementary file 1 [file nutrients-10-00254-s001.pdf]

Supplementary materials

Table S1. Statements within Health and Taste Attitudes Scales (HTAS) (Roininen et al., 1999)

	**Health-related subscales**	**Taste-related subscales**
Statements within each subscale	**GENERAL HEALTH INTEREST**	**CRAVING FOR SWEET FOODS**
	1 I am very particular about the healthiness of food.	1.R In my opinion it is strange that some people have cravings for chocolate.
	2 I always follow a healthy and balanced diet.	2.R In my opinion it is strange that some people have cravings for sweets.
	3 It is important for me that my diet is low in fat.	3.R In my opinion it is strange that some people have cravings for ice-cream.
	4 It is important for me that my daily diet contains a lot of vitamins and minerals.	4 I often have cravings for sweets.
	5.R* I eat what I like and I do not worry about healthiness of food.	5 I often have cravings for chocolate.
	6.R I do not avoid any foods, even if they may raise my cholesterol.	6 I often have cravings for ice-cream.
	7.R The healthiness of food has little impact on my food choices.	**USING FOOD AS A REWARD**
	8.R The healthiness of snacks makes no difference to me.	1 I reward myself by buying something really tasty.
	**LIGHT PRODUCT INTEREST**	2 I indulge myself by buying something really delicious.
	1.R In my opinion, the use of light products does not improve one's health.	3 When I am feeling down I want to treat myself with something really delicious.
	2.R I do not think that light products are healthier than conventional products.	4.R I avoid rewarding myself with food.
	3 I believe that eating light products keeps one's cholesterol level under control.	5.R In my opinion, comforting oneself by eating is self-deception.
	4.R In my opinion light products don't help to drop cholesterol levels.	6.R I try to avoid eating delicious food when I am feeling down.
	5 I believe that eating light products keeps one's body in good shape.	**PLEASURE**
	6 In my opinion by eating light products one can eat more without getting too many calories.	1.R I do not believe that food should always be a source of pleasure.
	**NATURAL PRODUCT INTEREST**	2.R The appearance of food makes no difference to me.
	1.R I do not care about additives in my daily diet.	3 It is important for me to eat delicious food on weekdays as well as weekends.
	2.R In my opinion, organically grown foods are no better for my health than those grown conventionally.	4 When I eat, I concentrate on enjoying the taste of food.
	3.R In my opinion, artificially flavoured foods are not harmful for my health.	5.R I finish my meal even when I do not like the taste of food.
	4 I try to eat foods that do not contain additives.	6 An essential part of my weekend is eating delicious food.
	5 I would like to eat only organically grown vegetables.	
	6 I do not eat processed foods, because I do not know what they contain.	*R (recoded answer)

Source: Roininen, K.; Lähteenmäki, L.; Tuorila, H. Quantification of consumer attitudes to health and hedonic characteristics of foods. Appetite 1999, 33, 71-88.

Table S2. Factor-loading matrix for the 4 major dietary patterns identified by principal component analysis, the GEBaHealth Study (*n* 1107)†

	Factor 1	Factor 2	Factor 3	Factor 4
	'Traditional Polish' DP	'Fruit & vegetables' DP	'Fast food & sweets' DP	'Dairy & fats' DP
White bread (including biscuits, muffins) frequency consumption[a]	0.65			
Meats, fish and eggs intake variety[b]	0.60			
Potatoes frequency consumption[a]	0.52			
Red meats frequency consumption[a]	0.51			
Margarine or butter frequency consumption[a]	0.45			0.45
Fats intake variety[b]	0.45			0.43
Fried chicken frequency consumption[a]	0.42			
Vegetables intake variety[b]		0.60		
Green salad frequency consumption[a]		0.57		
Fruit (without juices) frequency consumption[a]		0.55		
Prepared vegetables frequency consumption[a]		0.55		
Fruit intake variety[b]		0.54		
Beans frequency consumption[a]		0.45		
French fries or potato chips or corn chips or popcorn frequency consumption[a]			0.71	
Hamburgers or cheeseburgers frequency consumption[a]			0.60	
Ice cream frequency consumption[a]			0.52	
Doughnuts or pastries or cake or cookies frequency consumption[a]			0.50	
Sweets and snacks intake variety[b]			0.47	
Salad dressings or mayonnaise (not diet) frequency consumption[a]			0.42	
Cereals and potatoes intake variety[b]				0.56
Cheese or cheese spread frequency consumption[a]				0.54
Dairy products intake variety[b]				0.54
Whole milk frequency consumption[a]				0.49
Wholegrain bread frequency consumption[a]	-0.48			
Eigenvalues	4.36	2.39	1.68	1.44
Variance explained (%)[c]	14.5	9.0	5.6	4.8

†Factor loadings of ≤ |0.40| are not shown in the table for simplicity. Sorted by loadings from 1st to 4th factor. All data adjusted for survey weights. DP – dietary pattern.

[a] Food frequency consumption was expressed in points (range 0-4 points).

[b] Food intake variety was expressed in foods consumed per week, with ranges: 0-4 (food groups: dairy products; sweets and snacks), 0-6 (food groups: cereals and potatoes; fats; beverages), 0-8 (food group: fruit), 0-12 (food group: meats, fish and eggs), 0-14 (food group: vegetables).

[c] Total variance in dietary variables explained by 4 patterns is 33.9%

Table S3. Means with standard deviation (SD) for dietary characteristics across tertiles of dietary patterns, the GEBaHealth Study (*n* 1107)†

Dietary items	Total (*n* 1107)	'Traditional Polish' DP bottom tertile (*n* 367)	'Traditional Polish' DP middle tertile (*n* 364)	'Traditional Polish' DP upper tertile (*n* 376)	'Fruit & vegetables' DP bottom tertile (*n* 364)	'Fruit & vegetables' DP middle tertile (*n* 367)	'Fruit & vegetables' DP upper tertile (*n* 376)	'Fast foods & sweets' DP bottom tertile (*n* 365)	'Fast foods & sweets' DP middle tertile (*n* 365)	'Fast foods & sweets' DP upper tertile (*n* 377)	'Dairy and fats' DP bottom tertile (*n* 366)	'Dairy and fats' DP middle tertile (*n* 365)	'Dairy and fats' DP upper tertile (*n* 376)
White bread (including biscuits, muffins) frequency consumption[a]	3.1(1.3)	2.0(1.4)	3.3(0.9)	3.8(0.5)	3.4(1.0)	3.1(1.2)	2.7(1.4)	2.8(1.4)	3.1(1.1)	3.2(1.2)	2.6(1.5)	3.3(1.0)	3.4(1.0)
Meats, fish and eggs intake variety[b]	4.4(1.5)	3.4(1.4)	4.5(1.3)	5.3(1.2)	3.9(1.4)	4.4(1.5)	4.9(1.6)	4.4(1.5)	4.5(1.6)	4.4(1.6)	4.2(1.6)	4.4(1.5)	4.6(1.5)
Potatoes frequency consumption[a]	2.7(1.1)	2.0(1.2)	2.7(1.0)	3.3(0.8)	2.7(1.1)	2.7(1.1)	2.7(1.2)	2.5(1.2)	2.7(1.2)	2.9(1.0)	2.4(1.3)	2.8(1.0)	2.9(1.0)
Red meats frequency consumption[a]	1.3(1.1)	0.7(0.8)	1.2(0.9)	1.8(1.1)	1.1(1.1)	1.3(1.1)	1.4(1.1)	1.3(1.1)	1.3(1.1)	1.2(1.1)	1.2(1.0)	1.3(1.1)	1.3(1.0)
Margarine or butter frequency consumption[a]	3.4(1.1)	2.8(1.4)	3.6(0.8)	3.9(0.5)	3.5(0.9)	3.5(1.0)	3.2(1.3)	3.4(1.2)	3.4(1.1)	3.5(1.0)	2.9(1.5)	3.6(0.8)	3.8(0.6)
Fats intake variety[b]	3.5(1.4)	2.8(1.5)	3.5(1.2)	4.1(1.1)	3.3(1.4)	3.5(1.3)	3.6(1.4)	3.1(1.4)	3.6(1.3)	3.7(1.3)	2.8(1.5)	3.6(1.2)	4.1(1.1)
Fried chicken frequency consumption[a]	2.0(0.9)	1.5(1.0)	2.0(0.8)	2.3(0.7)	1.8(0.9)	2.0(0.9)	2.0(0.9)	1.9(0.9)	1.9(0.9)	2.0(0.9)	2.0(0.9)	2.0(0.9)	1.9(0.9)
Vegetables intake variety[b]	7.2(2.2)	7.0(2.2)	7.1(2.2)	7.6(2.2)	5.7(1.9)	7.2(1.8)	8.8(1.8)	7.5(2.2)	7.1(2.2)	7.1(2.3)	6.8(2.2)	7.2(2.3)	7.7(2.1)
Green salad frequency consumption[a]	2.1(1.1)	1.9(1.1)	1.9(1.0)	2.3(1.1)	1.4(0.9)	2.1(0.9)	2.7(0.9)	2.1(1.0)	2.0(1.1)	2.0(1.1)	2.2(1.1)	2.0(1.1)	2.0(1.0)
Fruit (without juices) frequency consumption[a]	2.5(1.1)	2.5(1.2)	2.4(1.1)	2.6(1.1)	1.8(1.0)	2.6(1.0)	3.2(0.8)	2.6(1.1)	2.5(1.1)	2.4(1.2)	2.3(1.1)	2.6(1.1)	2.6(1.1)
Prepared vegetables frequency consumption[a]	1.7(1.1)	1.7(1.1)	1.6(1.1)	1.8(1.1)	1.1(0.9)	1.6(1.0)	2.4(0.9)	1.7(1.1)	1.7(1.1)	1.7(1.1)	1.8(1.2)	1.7(1.0)	1.6(1.1)
Fruit intake variety[b]	3.6(1.6)	3.4(1.6)	3.6(1.6)	3.8(1.5)	2.8(1.3)	3.5(1.3)	4.6(1.5)	3.6(1.6)	3.7(1.5)	3.6(1.5)	3.2(1.5)	3.6(1.5)	4.0(1.6)
Beans frequency consumption[a]	0.7(0.8)	0.8(0.8)	0.7(0.8)	0.7(0.8)	0.3(0.6)	0.7(0.8)	1.2(0.9)	0.6(0.7)	0.7(0.8)	0.9(0.9)	0.8(0.9)	0.8(0.8)	0.7(0.8)
French fries or potato chips or corn chips or popcorn frequency consumption[a]	1.3(1.1)	1.0(1.0)	1.4(1.1)	1.5(1.1)	1.5(1.2)	1.3(1.1)	1.1(1.1)	0.4(0.6)	1.2(0.9)	2.2(1.0)	1.2(1.1)	1.4(1.1)	1.4(1.1)
Hamburgers or cheeseburgers frequency consumption[a]	0.3(0.7)	0.2(0.6)	0.4(0.7)	0.4(0.8)	0.3(0.6)	0.4(0.7)	0.4(0.7)	0.0(0.2)	0.2(0.4)	0.8(0.9)	0.4(0.8)	0.4(0.7)	0.3(0.6)
Ice cream frequency consumption[a]	0.6(0.9)	0.6(0.9)	0.6(0.9)	0.5(0.8)	0.6(0.9)	0.5(0.9)	0.6(0.8)	0.1(0.4)	0.5(0.7)	1.1(1.1)	0.5(0.8)	0.6(0.9)	0.6(0.8)
Doughnuts or pastries or cake or cookies frequency consumption[a]	1.9(1.1)	1.6(1.2)	2.0(1.1)	2.0(1.1)	1.9(1.1)	1.9(1.1)	1.8(1.1)	1.2(1.0)	1.9(1.1)	2.5(1.0)	1.5(1.1)	1.9(1.1)	2.2(1.1)
Sweets and snacks intake variety[b]	3.1(1.0)	2.7(1.1)	3.1(0.9)	3.4(0.8)	3.1(0.9)	3.1(1.0)	2.9(1.1)	2.4(1.1)	3.1(0.9)	3.6(0.6)	2.7(1.1)	3.2(1.0)	3.4(0.8)
Salad dressings or mayonnaise (not diet) frequency consumption[a]	1.3(1.1)	1.2(1.1)	1.4(1.1)	1.5(1.2)	1.1(1.1)	1.4(1.1)	1.5(1.1)	0.7(0.9)	1.4(1.1)	1.9(1.1)	1.2(1.1)	1.3(1.1)	1.5(1.1)
Cereals and potatoes intake variety[b]	3.7(1.1)	3.5(1.3)	3.8(1.0)	3.8(1.0)	3.4(1.0)	3.8(1.1)	3.9(1.1)	3.8(1.1)	3.7(1.1)	3.7(1.1)	3.0(1.0)	3.7(0.9)	4.4(0.9)
Cheese or cheese spread frequency consumption[a]	2.5(1.1)	2.5(1.1)	2.5(1.1)	2.4(1.1)	2.4(1.2)	2.5(1.1)	2.5(1.1)	2.3(1.1)	2.4(1.1)	2.7(1.1)	1.8(1.1)	2.4(0.9)	3.1(0.9)
Dairy products intake variety[b]	2.3(1.0)	2.4(1.0)	2.3(1.0)	2.4(1.0)	2.1(1.0)	2.3(1.0)	2.6(0.9)	2.3(1.0)	2.2(1.0)	2.5(1.0)	1.7(1.0)	2.4(0.8)	2.9(0.7)
Whole milk frequency consumption[a]	1.9(1.5)	1.7(1.5)	2.0(1.5)	2.0(1.5)	2.0(1.4)	2.0(1.5)	1.8(1.5)	1.8(1.5)	2.0(1.5)	2.0(1.4)	1.0(1.3)	2.0(1.4)	2.7(1.3)
Wholegrain bread frequency consumption[a]	1.5(1.5)	2.5(1.5)	1.4(1.4)	0.7(1.1)	0.7(1.2)	1.6(1.5)	2.1(1.5)	1.6(1.6)	1.5(1.5)	1.4(1.5)	1.3(1.5)	1.4(1.4)	1.7(1.6)
Beverages (without alcohols) intake variety[b]	3.5(1.1)	3.4(1.1)	3.6(1.1)	3.6(1.1)	3.2(1.0)	3.5(1.1)	3.8(1.0)	3.2(1.1)	3.5(1.1)	3.8(1.0)	3.4(1.1)	3.5(1.1)	3.7(1.1)
Eggs frequency consumption[a]	2.0(0.9)	1.8(1.0)	1.9(0.9)	2.3(0.8)	1.8(0.9)	2.0(0.9)	2.3(0.9)	2.0(0.9)	2.1(0.9)	2.0(1.0)	1.9(0.9)	1.9(0.9)	2.3(0.9)
Fruit or vegetable juices frequency consumption[a]	1.7(1.2)	1.8(1.3)	1.8(1.2)	1.5(1.2)	1.2(1.1)	1.7(1.2)	2.1(1.2)	1.4(1.1)	1.8(1.2)	1.9(1.2)	1.7(1.2)	1.7(1.2)	1.7(1.2)
Hot dogs or frankfurters frequency consumption[a]	1.2(1.1)	0.7(0.9)	1.2(1.1)	1.7(1.1)	1.1(1.1)	1.2(1.1)	1.3(1.1)	0.8(0.9)	1.2(1.0)	1.7(1.1)	1.2(1.1)	1.3(1.1)	1.1(1.1)
High-fiber or bran cereal frequency consumption[a]	1.1(1.2)	1.6(1.3)	1.0(1.2)	0.6(1.0)	0.6(0.9)	1.1(1.2)	1.6(1.3)	1.2(1.3)	1.0(1.2)	1.0(1.2)	0.7(1.0)	0.9(1.0)	1.7(1.4)
Luncheon meats or bacon or fatty sausages frequency consumption[a]	0.5(0.9)	0.2(0.6)	0.5(0.8)	0.9(1.0)	0.4(0.8)	0.6(0.9)	0.6(0.9)	0.2(0.6)	0.5(0.8)	0.8(1.1)	0.6(0.9)	0.6(0.9)	0.4(0.8)

†All data adjusted for survey weights. DP – dietary pattern.

[a] Food frequency consumption was expressed in points (range 0-4 points).

[b] Food intake variety was expressed in foods consumed per week, with ranges from: 0-4 (food groups: dairy products; sweets and snacks), 0-6 (food groups: cereals and potatoes; fats; beverages), 0-8 (food group: fruit), 0-12 (food group: meats, fish and eggs), 0-14 (food group: vegetables).

Table S4. Sample distribution (%) of dietary patterns depending on attitudes towards health and taste in girls and young women, the GEBaHealth Study (*n* 1107)†

Attitudes towards health and taste	*n*	'Traditional Polish' DP			*P*	'Fruit & vegetables' DP			*P*	'Fast food & sweets' DP			*P*	'Dairy & fats' DP			*p*
		bottom tertile (*n* 367)	middle tertile (*n* 364)	upper tertile (*n* 376)		bottom tertile (*n* 364)	middle tertile (*n* 367)	upper tertile (*n* 376)		bottom tertile (*n* 365)	middle tertile (*n* 365)	upper tertile (*n* 377)		bottom tertile (*n* 366)	middle tertile (*n* 365)	upper tertile (*n* 376)	
**General health interest**	1107				****				****				****				***
negative	123	14.9	40.1	45.0		50.3	33.1	16.6		14.7	30.4	54.9		36.8	32.9	30.3	
neutral	810	31.1	34.3	34.6		34.8	33.3	31.9		31.5	34.2	34.3		29.9	35.4	34.7	
positive	174	55.3	21.4	23.3		11.4	32.7	55.9		52.8	29.5	17.7		45.1	21.5	33.4	
**Light product interest**					ns				ns				ns				ns
negative	222	39.4	27.0	33.6		35.1	27.4	37.5		37.4	32.2	30.4		35.6	32.2	32.2	
neutral	829	31.0	34.3	34.7		32.6	34.7	32.7		31.5	33.7	34.8		32.4	32.9	34.7	
positive	56	39.7	35.4	24.9		28.4	32.5	39.1		36.9	26.6	36.5		32.2	36.7	31.1	
**Natural product interest**					**				***				****				ns
negative	48	18.9	44.7	36.4		47.0	30.6	22.4		8.4	23.4	68.2		32.6	31.7	35.7	
neutral	828	31.7	32.7	35.6		35.1	33.3	31.6		31.1	34.4	34.5		33.7	33.6	32.7	
positive	231	41.2	31.0	27.8		22.1	33.3	44.6		44.8	30.0	25.2		30.8	30.7	38.5	
**Craving for sweet foods**					**				ns				****				**
negative	69	42.2	30.6	27.2		29.5	37.3	33.2		45.4	37.5	17.1		45.0	36.8	18.2	
neutral	497	36.6	34.1	29.3		32.4	30.6	37.0		38.3	31.7	30.0		35.3	33.5	31.2	
positive	541	28.8	32.1	39.1		33.8	35.0	31.2		26.6	33.6	39.8		29.5	31.9	38.6	
**Using food as reward**					ns				ns				****				***
negative	409	37.7	30.7	31.6		31.4	31.7	36.9		44.2	34.5	21.3		38.2	32.1	29.7	
neutral	606	30.6	34.0	35.4		33.4	33.4	33.2		27.9	32.0	40.1		31.1	34.9	34.0	
positive	92	29.5	35.8	34.7		36.3	38.2	25.5		16.7	33.2	50.1		23.1	23.8	53.1	
**Pleasure**					ns				**				ns				**
negative	14	56.4	26.9	16.7		44.2	45.2	10.6		30.4	45.7	23.9		67.4	14.2	18.4	
neutral	771	33.2	33.0	33.8		35.9	30.7	33.4		33.7	33.0	33.3		32.9	35.5	31.6	
positive	322	31.8	33.1	35.1		25.2	38.6	36.2		31.3	32.4	36.3		32.1	27.5	40.4	

†All data adjusted for survey weights. DP – dietary pattern. Statistically significant (chi^2 test): **P<0.01, ***P<0.001, ****P<0.0001; ns - statistically insignificant

Table S5. Sample distribution (%) of dietary patterns depending on attitudes towards health and taste in girls and young women across BMI categories, the GEBaHealth Study (*n* 1092)†

Attitudes towards health and taste	*n*	**Underweight females‡ (*n* 110)**															
		'Traditional Polish' DP			*P*	'Fruit & vegetables' DP			*P*	'Fast food & sweets' DP			*P*	'Dairy & fats' DP			*P*
		bottom tertile (*n* 26)	middle tertile (*n* 35)	upper tertile (*n* 49)		bottom tertile (*n* 39)	middle tertile (*n* 34)	upper tertile (*n* 37)		bottom tertile (*n* 32)	middle tertile (*n* 25)	upper tertile (*n* 53)		bottom tertile (*n* 29)	middle tertile (*n* 43)	upper tertile (*n* 38)	
**General health interest**	110				*				*				ns				ns
negative	14	3.8	39.6	56.5		67.9	21.8	10.3		17.8	15.8	66.4		42.6	47.2	10.1	
neutral	83	23.2	35.9	40.8		34.0	29.0	37.0		27.8	22.8	49.4		26.6	36.6	36.8	
positive	13	44.5	0.0	55.5		6.0	52.4	41.6		51.2	27.9	21.0		9.8	41.3	49.0	
**Light product interest**					ns				ns				ns				ns
negative	23	21.7	28.9	49.5		28.3	22.3	49.3		15.3	34.9	49.8		29.9	43.4	26.7	
neutral	83	22.2	34.9	42.9		37.8	33.2	29.0		34.3	17.9	47.8		27.3	37.7	35.0	
positive	4	49.2	0.0	50.8		19.8	31.0	49.2		9.7	41.1	49.2		0.0	31.0	69.0	
**Natural product interest**					ns				*				ns				ns
negative	4	0.0	63.0	37.0		100.0	0.0	0.0		0.0	0.0	100.0		17.3	63.0	19.7	
neutral	84	19.9	32.8	47.4		38.4	27.7	33.8		27.8	25.5	46.7		30.9	35.5	33.6	
positive	22	39.7	24.8	35.5		11.8	47.5	40.6		39.8	14.6	45.6		12.6	45.9	41.5	
**Craving for sweet foods**					ns				ns				ns				ns
negative	7	20.9	54.3	24.8		52.4	14.2	33.5		63.5	26.8	9.8		33.8	36.9	29.3	
neutral	42	26.6	28.7	44.8		19.1	39.2	41.7		31.0	24.1	44.9		27.4	32.7	39.8	
positive	61	21.2	31.8	46.9		44.0	27.1	28.9		23.9	20.8	55.3		25.3	42.8	31.9	
**Using food as reward**					ns				ns				ns				ns
negative	34	28.8	24.8	46.4		43.6	36.2	20.1		36.6	28.0	35.4		39.1	33.7	27.2	
neutral	68	22.2	32.9	44.9		29.2	29.8	41.0		27.0	19.3	53.6		20.5	43.5	35.9	
positive	8	8.6	56.1	35.2		48.0	17.6	34.3		17.4	25.5	57.1		26.4	17.5	56.2	
**Pleasure**					ns				ns				ns				ns
negative	0	0.0	0.0	0.0		0.0	0.0	0.0		0.0	0.0	0.0		0.0	0.0	0.0	
neutral	71	23.7	35.9	40.4		33.1	30.4	36.5		32.4	20.4	47.1		23.9	38.6	37.5	
positive	39	22.4	25.3	52.2		38.6	31.7	29.6		23.5	26.1	50.3		31.7	38.5	29.8	

†All data adjusted for survey weights; total BMI sample size is smaller due to missing data (*n* 1092). DP – dietary pattern. ‡BMI categories determined according to IOTF standards [28], i.e. for girls 13-18 years old according to age-sex-specific BMI cut-offs and for girls >18 years old according to cut-offs for girls at age 18). Statistically significant (chi^2 test): *P<0.05; ns - statistically insignificant

Table S5. *Cont.*†

Attitudes towards health and taste	*n*	'Traditional Polish' DP			*P*	'Fruit & vegetables' DP			*P*	'Fast food & sweets' DP			*P*	'Dairy & fats' DP			*P*
**Normal weight females‡ (*n* 849)**		bottom tertile (*n* 295)	middle tertile (*n* 276)	upper tertile (*n* 278)		bottom tertile (*n* 296)	middle tertile (*n* 287)	upper tertile (*n* 266)		bottom tertile (*n* 268)	middle tertile (*n* 293)	upper tertile (*n* 288)		bottom tertile (*n* 271)	middle tertile (*n* 278)	upper tertile (*n* 300)	
**General health interest**	849				****				****				****				*
negative	92	18.8	42.1	39.1		53.2	37.6	9.2		13.1	32.6	54.3		34.6	31.3	34.1	
neutral	631	32.8	32.8	34.4		37.0	33.2	29.9		31.3	34.6	34.1		29.3	34.6	36.1	
positive	126	56.2	23.7	20.0		11.4	33.9	54.7		46.7	34.8	18.4		43.0	24.7	32.2	
**Light product interest**					ns				ns				ns				ns
negative	167	39.9	26.5	33.7		39.3	29.3	31.4		36.2	32.2	31.6		33.4	31.2	35.4	
neutral	641	32.8	33.8	33.4		34.4	35.0	30.6		30.1	35.3	34.6		31.7	32.6	35.8	
positive	41	45.8	35.3	18.9		25.5	32.6	42.0		37.2	30.0	32.8		30.1	42.2	27.7	
**Natural product interest**					*				***				****				ns
negative	38	16.4	50.6	33.0		44.0	35.3	20.7		1.2	25.9	72.9		28.1	32.2	39.7	
neutral	638	34.7	31.5	33.8		37.4	33.9	28.7		31.0	35.1	33.9		32.7	33.1	34.2	
positive	173	39.2	31.9	28.9		23.9	32.8	43.3		40.4	34.1	25.5		29.9	31.8	38.3	
**Craving for sweet foods**					***				ns				***				**
negative	51	45.6	26.3	28.0		25.6	42.5	31.8		41.4	38.6	20.0		47.1	34.4	18.5	
neutral	382	38.7	35.1	26.3		36.5	29.1	34.4		37.6	32.1	30.2		35.1	33.7	31.3	
positive	416	29.9	30.8	39.3		34.6	37.0	28.5		24.9	36.1	39.0		27.2	31.7	41.1	
**Using food as reward**					ns				ns				****				***
negative	321	37.3	31.6	31.1		31.6	32.5	35.9		43.3	35.7	21.0		35.2	33.3	31.5	
neutral	457	32.7	33.0	34.3		36.5	33.5	30.0		25.9	33.4	40.7		31.5	34.1	34.4	
positive	71	37.1	33.0	29.9		39.1	41.3	19.6		15.6	35.8	48.6		20.2	21.4	58.4	
**Pleasure**					ns				**				ns				**
negative	10	77.2	0.0	22.8		55.2	30.4	14.4		33.7	33.6	32.7		63.3	19.5	17.2	
neutral	603	34.3	32.6	33.0		38.2	30.8	31.0		32.6	34.0	33.5		31.9	35.9	32.2	
positive	236	34.3	33.3	32.4		25.7	41.5	32.8		29.1	35.7	35.1		30.7	25.4	43.9	

†All data adjusted for survey weights; total BMI sample size is smaller due to missing data (*n* 1092). DP – dietary pattern. ‡BMI categories determined according to IOTF standards [28], i.e. for girls 13-18 years old according to age-sex-specific BMI cut-offs and for girls >18 years old according to cut-offs for girls at age 18). Statistically significant (chi² test): *P<0.05, **P<0.01, ***P<0.001, ****P<0.0001; ns - statistically insignificant

Table S5. *Cont.*†

Attitudes towards health and taste	*n*	Overweight females‡ (*n* 133)															
		'Traditional Polish' DP				'Fruit & vegetables' DP				'Fast food & sweets' DP				'Dairy & fats' DP			
		bottom tertile (*n* 41)	middle tertile (*n* 49)	upper tertile (*n* 43)	*P*	bottom tertile (*n* 25)	middle tertile (*n* 43)	upper tertile (*n* 65)	*P*	bottom tertile (*n* 59)	middle tertile (*n* 43)	upper tertile (*n* 31)	*P*	bottom tertile (*n* 57)	middle tertile (*n* 40)	upper tertile (*n* 36)	*P*
**General health interest**	133				***				ns				***				**
negative	16	3.8	32.4	63.7		22.3	20.0	57.7		22.6	33.8	43.7		38.6	32.1	29.3	
neutral	84	24.9	44.2	30.9		20.1	39.9	40.0		34.8	40.8	24.4		34.3	41.3	24.5	
positive	33	56.8	21.5	21.7		12.1	19.8	68.1		77.0	11.0	12.0		66.4	2.2	31.4	
**Light product interest**					ns				ns				ns				ns
negative	31	47.2	29.8	23.0		14.5	22.3	63.2		57.2	31.8	11.0		48.4	31.0	20.6	
neutral	92	27.0	37.3	35.7		16.5	36.8	46.7		39.0	35.2	25.8		39.0	31.9	29.1	
positive	10	7.8	59.2	33.0		48.8	24.4	26.8		48.8	7.8	43.4		62.4	13.3	24.3	
**Natural product interest**					**				ns				**				ns
negative	4	38.8	0.0	61.2		20.1	38.8	41.1		58.9	41.1	0.0		41.1	20.1	38.8	
neutral	95	22.4	40.3	37.3		18.3	34.7	47.1		33.9	36.4	29.7		41.6	35.8	22.6	
positive	34	52.0	32.1	16.0		18.5	25.7	55.8		70.9	20.4	8.7		46.6	16.2	37.3	
**Craving for sweet foods**					ns				ns				ns				ns
negative	10	40.3	34.7	25.0		32.5	27.9	39.6		52.0	40.4	7.6		42.4	49.5	8.1	
neutral	69	31.0	33.4	35.6		17.8	33.7	48.5		44.9	35.3	19.8		38.5	34.4	27.1	
positive	54	27.7	42.3	30.0		16.4	31.8	51.7		41.3	27.4	31.3		48.5	21.7	29.8	
**Using food as reward**					*				ns				*				ns
negative	49	44.3	29.2	26.5		18.5	22.7	58.8		55.5	30.4	14.1		56.6	22.0	21.3	
neutral	71	26.1	42.4	31.5		19.3	39.0	41.7		39.8	35.6	24.6		34.2	34.2	31.7	
positive	13	0.0	38.3	61.7		12.7	34.0	53.3		22.2	23.1	54.6		37.6	41.7	20.7	
**Pleasure**					ns				ns				ns				ns
negative	4	0.0	100.0	0.0		14.4	85.6	0.0		21.5	78.5	0.0		78.5	0.0	21.5	
neutral	87	32.7	33.2	34.1		21.4	30.1	48.4		41.0	37.5	21.5		43.5	32.2	24.3	
positive	42	28.2	39.6	32.1		12.4	32.7	54.9		52.0	18.2	29.8		38.3	29.3	32.4	

†All data adjusted for survey weights; total BMI sample size is smaller due to missing data (*n* 1092). DP – dietary pattern. ‡BMI categories determined according to IOTF standards [28], i.e. for girls 13-18 years old according to age-sex-specific BMI cut-offs and for girls >18 years old according to cut-offs for girls at age 18). Statistically significant (chi² test): *$P<0.05$, **$P<0.01$, ***$P<0.001$; ns - statistically insignificant
